# Airway obstruction due to a laryngeal polyp following insertion of a laryngeal mask airway

**DOI:** 10.1186/s40981-018-0180-3

**Published:** 2018-06-05

**Authors:** Satoshi Fuseya, Takashi Ichino, Satoshi Tanaka, Kumiko Ishida, Takashi Ishida, Mikito Kawamata

**Affiliations:** Department of Anesthesiology and Resuscitology, Shinshu University School of Medicine, 3-1-1 Asahi, Matsumoto City, Nagano 390-8621 Japan

**Keywords:** Airway obstruction, Laryngeal polyp, Laryngeal mask airway

## Abstract

**Background:**

Laryngeal mask airway (LMA) insertion contributes to airway protection in patients with a laryngeal tumor around the glottis. There has been no report of LMA insertion itself exacerbating airway obstruction in such patients.

**Case presentation:**

A 62-year-old male underwent elective surgical resection of a large laryngeal polyp. The polyp was attached to the right vocal fold and synchronously swung inward into the trachea and outward to the larynx with inspiration and expiration, respectively. Although manual positive pressure ventilation was easily achieved without any airway obstruction after anesthetic induction, the airway was completely obstructed by the polyp lodged between the vocal cords immediately after LMA insertion. Soon after removal of the LMA, patency of the airway was dramatically improved.

**Conclusion:**

Our experience indicates that we should pay attention to airway obstruction due to lodging of the polyp between the vocal cords after LMA insertion in patients with a laryngeal polyp.

## Background

Surgery and anesthetic management of patients with a laryngeal tumor occupying the space of the glottis is challenging for anesthesiologists because airway obstruction can occur during surgery. Temporary tracheostomy, endotracheal intubation using a thin endotracheal tube (ETT), and high-frequency jet ventilation have been used for airway management for laryngoscopic surgery with a rigid laryngoscope in such patients [1]. Another option is a laryngeal mask airway (LMA), which has been used for airway protection, and introduction of a flexible laryngoscope for surgery has been successfully used in such patients [2]. There has been no report of LMA insertion itself exacerbating airway obstruction in such patients. Here, we report a patient with a laryngeal polyp in whom manual positive pressure ventilation was easily achieved after anesthetic induction, but the airway was completely obstructed by the polyp lodged between the vocal cords following LMA insertion.

## Case presentation

**Fig. 1:**
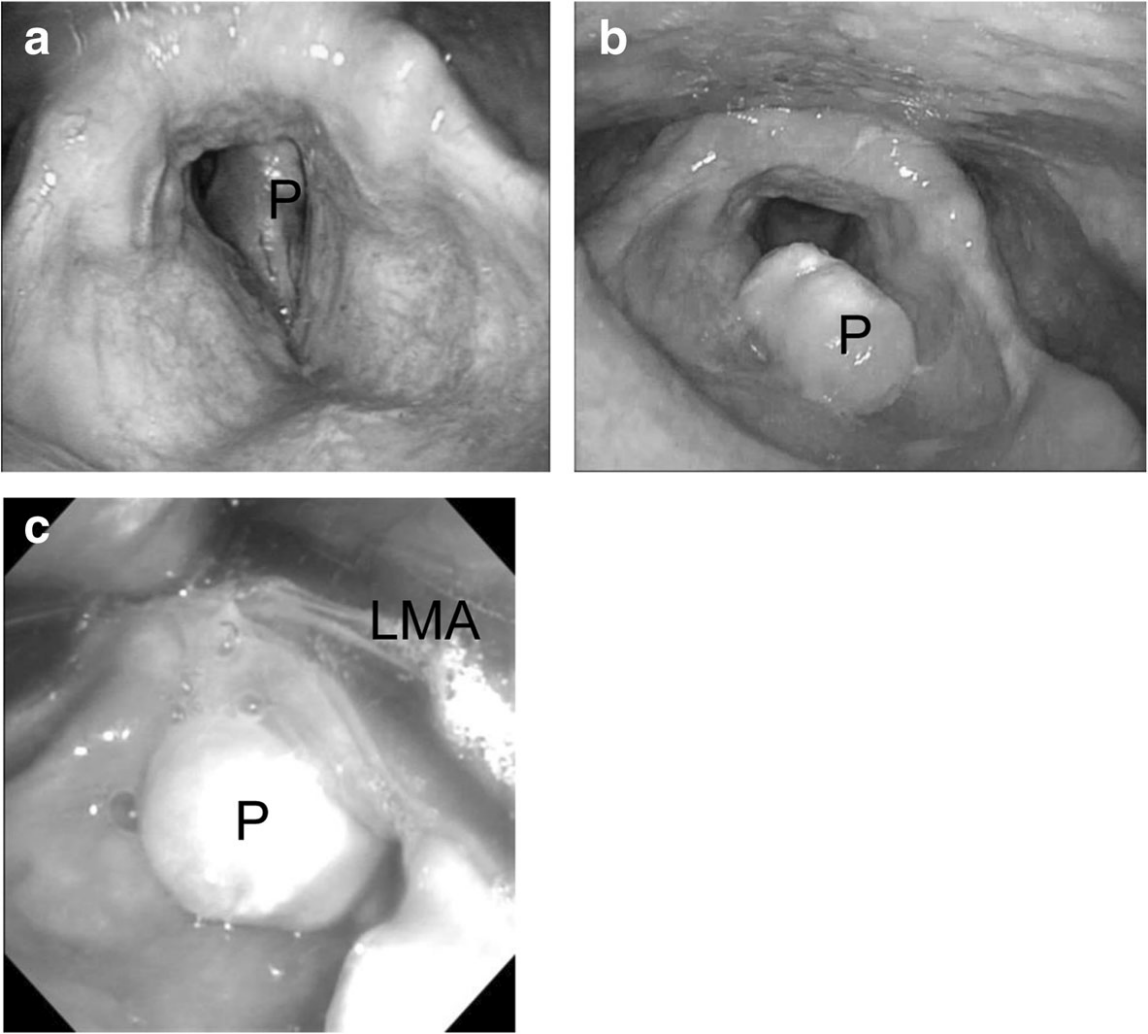
Laryngoscopic images of the glottis in a patient with a large laryngeal polyp. Preoperative laryngoscopic images of the glottis show the large laryngeal polyp attached to the right vocal fold that synchronously swung inward into the trachea and outward to the larynx with inspiration (**a**) and expiration (**b**), respectively. An intraoperative image shows that the polyp was located between the vocal cords and immovable after LMA insertion (**c**). P laryngeal polyp, LMA laryngeal mask airway

A 62-year-old male weighing 75 kg and with a height of 162 cm complained of discomfort in the throat. Preoperative laryngoscopy revealed a large laryngeal polyp attached to the right vocal fold that synchronously swung inward into the trachea and outward to the larynx with inspiration and expiration, respectively (Fig. 1a, b). The space between the vocal cords was too narrow due to the presence of the polyp for an endotracheal tube to be inserted. In addition, a surgical procedure could not be performed if the polyp moved into the trachea after endotracheal intubation. Thus, we decided to reduce the volume of the polyp by using a flexible bronchoscope through the LMA and then to perform total removal of the polyp by using a rigid laryngoscope with endotracheal intubation. If the airway did not remain patent by such airway management, we planned to perform temporary tracheostomy for removal of the polyp using a rigid laryngoscope.

The patient was not given premedication, and routine noninvasive monitoring including blood pressure, percutaneous oxygen saturation (SpO_2_), and end-tidal CO_2_ was performed in the operating room. SpO_2_ was 97% on room air, and oxygen was delivered by a facemask at 7 L/min in the operating room. During anesthesia that was induced with incremental administration of propofol (total dose of 170 mg), spontaneous ventilation was manually assisted and was followed by manual positive pressure ventilation without any airway obstruction. However, complete airway obstruction occurred immediately after a size 4 LMA (LMA Supreme™, Teleflex Co., NC, USA) was inserted. Maneuvers including changing the LMA position and increasing and decreasing the volume of cuff air did not improve the difficult airway. A flexible laryngoscope revealed airway obstruction due to the polyp lodged between the vocal cords (Fig. 1c), and the polyp could not be moved by pulling with forceps through the LMA. Spontaneous respiration returned, but the airway was still not patent even though the maneuvers were repeatedly tried. SpO_2_ decreased to 88%, and the LMA was removed. Soon after the removal, patency of the airway was dramatically improved and SpO_2_ returned to 100%. Transient tracheostomy was then carried out under general anesthesia with 2.0% of sevoflurane in 40% oxygen with assisted spontaneous ventilation and injection of 250 μg fentanyl. Resection of the polyp was successfully performed using a rigid laryngoscope. The tracheostomy was closed on postoperative day 5, and the patient was discharged on postoperative day 7.

## Discussion

Airway management for laryngoscopic surgery in patients with a laryngeal tumor includes temporary tracheostomy, endotracheal intubation using a thin ETT, transglottic or intercricoid high-frequency jet ventilation, and LMA insertion [1, 3]. However, it has been reported that the polyp was dislodged by the ETT and airway obstruction occurred when the trachea was intubated [4]. When high-frequency jet ventilation is performed, there are risks of complications such as subcutaneous emphysema and pneumothorax [3]. An LMA has been used for airway protection, and introduction of a flexible laryngoscope via the LMA has been successfully used in patients with a laryngeal polyp [1, 5]. Thus, we considered that LMA insertion could avoid invasive tracheostomy and would allow surgery to be performed using a flexible bronchoscope.

However, it has been reported that LMA insertion itself can cause deformity of the vocal cords [2], possibly resulting in airway obstruction due to lodging of the polyp between the vocal cords as in our case. LMA insertion may thus exacerbate airway obstruction in patients with a laryngeal polyp in the glottis, and caution should be paid for LMA insertion in such patients. In our case, the polyp was smoothly swung inward into the trachea in inspiration and swung outward to the larynx in expiration through the vocal cords during spontaneous respiration without dyspnea. Accordingly, reduction of the volume of the polyp by using a flexible laryngoscope under mask ventilation with light sedation might have been another option. However, this procedure may also have the risk of unanticipated difficult airway due to accidental deep sedation, bleeding from the tumor and abrupt body movement caused by surgical intervention.

LMA insertion enables successful ventilation in a patient with airway obstruction due to several large laryngeal polyps [5]. Thus, the use of an LMA contributes to the relief of airway obstruction in some patients with a large laryngeal polyp. However, LMA insertion also has a potential risk of exacerbating airway obstruction in such patients as shown in our case. Thus, when laryngoscopic surgery with a flexible laryngoscope through an LMA is planned in patients with laryngeal lesions, preparation should also be made for transcricoid jet ventilation and tracheostomy prior to surgery.

## Conclusion

In conclusion, our experience indicates that we should pay attention to airway obstruction due to lodging of the polyp between the vocal cords after LMA insertion in patients with a laryngeal polyp.

## Abbreviations

ETT: Endotracheal tube
LMA: Laryngeal mask airway
SpO_2_: Percutaneous oxygen saturation
